# 非小细胞肺癌脑转移高危因素研究进展

**DOI:** 10.3779/j.issn.1009-3419.2022.101.08

**Published:** 2022-03-20

**Authors:** 爽 孙, 玉 门, 周光 惠

**Affiliations:** 1 100021 北京，国家癌症中心/国家肿瘤临床医学研究中心/中国医学科学院，北京协和医学院肿瘤医院放疗科 Department of Radiation Oncology, National Cancer Center/National Clinical Research Center for Cancer/Cancer Hospital, Chinese Academy of Medical Sciences and Peking Union Medical College, Beijing 100021, China; 2 100021 北京，国家癌症中心/国家肿瘤临床医学研究中心/中国医学科学院，特需医疗部 Department of VIP Medical Services, National Cancer Center/National Clinical Research Center for Cancer/Cancer Hospital, Chinese Academy of Medical Sciences and Peking Union Medical College, Beijing 100021, China

**Keywords:** 肺肿瘤, 脑转移, 预防性脑照射, Lung neoplasms, Brain metastasis, Prophylactic cranial irradiation

## Abstract

非小细胞肺癌（non-small cell lung cancer, NSCLC）脑转移是常见的治疗失败模式，发生脑转移的NSCLC患者中位生存时间仅为1个月-2个月。预防性脑照射（prophylactic cranial irradiation, PCI）可延缓脑转移的发生，但对NSCLC患者的生存获益仍存在争议，因此，通过预测脑转移的风险筛选可能从PCI中获益的人群尤为重要。本文就NSCLC脑转移的高危因素进行综述。

## Abstract (en)

Brain metastasis of non-small cell lung cancer (NSCLC) is a common treatment failure mode, and the median survival time of NSCLC patients with brain metastasis is only 1 mon-2 mon.Prophylactic cranial irradiation (PCI) can delay the occurrence of brain metastasis, but the survival benefits of NSCLC patients are still controversial.It is particularly important to identify the patients who are most likely to benefit from PCI.This article reviews the high risk factors of brain metastasis in NSCLC.

脑转移不仅是非小细胞肺癌（non-small cell lung cancer, NSCLC）初诊时的常见转移部位（10%-20%），在NSCLC治疗后亦有高达30%-54%的NSCLC患者会发生脑转移。脑转移患者预后较差，中位生存时间（median survival time, MST）仅为1个月-2个月，且严重影响患者生活质量^[1-3]^。虽然近年来，多学科治疗改善了脑转移患者生存，但整体疗效并不理想。全脑放疗被广泛应用于NSCLC脑转移治疗，然而MST仅为3个月-6个月；手术或立体定向放射治疗可有效控制NSCLC脑转移，但MST也仅可延长至9个月-10个月^[4-9]^。因此，预防NSCLC脑转移的发生可能是改善NSCLC患者生存的重要环节。预防性脑照射（prophylactic cranial irradiation, PCI）可将脑转移的发生率降低50%，是减缓NSCLC患者脑转移最有效的方法。然而多项研究^[10-12]^报道，不加选择地对NSCLC患者进行PCI并不能延长MST，且部分患者不能耐受PCI带来的副反应。这表明不是所有患者都可从PCI中获益，因而筛选NSCLC脑转移高危患者具有重要意义。本文对NSCLC脑转移高危因素进行综述。

## 1 临床相关因素

既往多数研究报道的NSCLC脑转移危险因素包括：年龄、肿瘤标记物水平、病理组织学、分期、治疗方式等。

### 1.1 年龄

Hubbs等^[13]^对975例I期-II期NSCLC患者进行多因素分析，结果显示年轻的患者更易发生脑转移（HR=1.03, *P* < 0.01）。同样地，Ding等^[14]^回顾性分析217例pIIIa-N2期NSCLC患者，发现年龄≤60岁及年龄 > 60岁的患者5年脑转移率分别为37.9%和24.9%（*P*=0.035）。Goncalves等^[15]^提出NSCLC的脑转移在80岁或以上人群中最低（3%），在20岁-39岁人群中相对较高（19%）。这可能是年轻患者的肿瘤生物学行为较差或者年轻患者微血管生成较好的原因造成的。另外几项研究^[16-18]^得到不同结论，即年龄不是脑转移的危险因素。年龄是否为NSCLC脑转移的高危因素仍需进一步探索。

### 1.2 肿瘤标记物

#### 1.2.1 癌胚抗原（carcinoembryonic antigen, CEA）

CEA作为肺癌患者的预后和预测指标，其应用一直存在争论。多项文献^[19, 20]^报道，NSCLC患者术前血清CEA水平与术后复发呈正相关，但CEA与脑转移的关系尚不明确。Liang等^[18]^纳入了193例IIIa期NSCLC患者，发现CEA升高是脑转移的独立危险因素（HR=2.152, 95%CI: 1.169-3.963, *P*=0.014）。周开甲等^[16]^也得到了相同的结论，治疗前血清CEA > 10.0 μg/L的患者更易发生脑转移（HR=2.083, 95%CI: 1.174-3.965, *P*=0.031）。Arrieta等^[21]^通过前瞻性研究分析了293例IIIb期-IV期NSCLC患者，发现CEA浓度≥40 ng/mL是脑转移的独立危险因素（RR=11.4, 95%CI: 1.7-74, *P* < 0.01）。以上结果均提示NSCLC患者血清CEA升高是脑转移的独立预测因素。

#### 1.2.2 神经元特异性烯醇化酶（neuron-specific enolase, NSE）

NSE主要在神经内分泌肿瘤中表达，在NSCLC中仅有10%的患者表达NSE，但近期多项研究关注NSE水平在NSCLC脑转移预测中的作用。Zhang等^[22]^发现高水平NSE（> 60.00 ng/mL）患者发生脑转移的概率分别是中水平（30.00 ng/mL-60.00 ng/mL）、低水平（0.00 ng/mL-30.00 ng/mL）的2.17倍（*P*=0.062）、9.52倍（*P* < 0.001）。血清NSE能否作为NSCLC患者脑转移的预测指标仍有待研究。

### 1.3 T、N分期

T、N分期一直被认为是影响包括脑转移在内的NSCLC复发的独立危险因素。有研究^[23]^回顾性分析1, 218例NSCLC患者的资料，发现pT分期较晚患者脑转移的发生率明显增高（pT1 *vs* pT2-4: 4.5% *vs* 8.1%, OR=1.80, *P*=0.035），pN分期也与脑转移显著相关（pN0-1 *vs* pN2-3: 5.2% *vs* 13.8%, OR=2.66, *P* < 0.001)。有研究^[24]^显示T分期晚的NSCLC患者脑转移风险明显增加，T2、T3、T4较T1患者脑转移发生风险分别增加了1.856倍、2.347倍和3.464倍（*P*=0.006）。更多的研究关注N分期对NSCLC脑转移的影响。Hubbs等^[13]^的研究表明，转移淋巴结是脑转移的预测因素，肺门淋巴结受累的患者更容易发生脑转移（HR=1.18, *P*=0.04）。另一项研究^[24]^也得出相似的结论，与N0患者相比，N1、N2患者脑转移风险分别增加3.332倍、4.935倍。Zhang等^[22]^发现4个以上淋巴结转移的NSCLC患者发生脑转移的风险是无淋巴结转移患者的3.90倍（95%CI: 2.09-7.28, *P* < 0.001）。Ding等^[14]^分析了217例经根治性手术治疗的IIIa-N2期NSCLC患者，发现转移淋巴结占检测淋巴结（lymph node ratio, LNR）≥30%的患者术后脑转移风险明显升高（RR=2.35, 95%CI: 1.10-5.02, *P*=0.027），LNR≥30%的鳞状细胞癌患者中，1年、3年和5年累积脑转移率分别为9.2%、18.5%和18.5%；而在非鳞状细胞癌患者中，1年、3年和5年累积脑转移率可高至18.1%、48.6%和57.3%。以上研究总体显示T分期、N分期是NSCLC患者脑转移的预测因素，T分期、N分期越高越易发生脑转移。

### 1.4 病理

NSCLC可进一步分为鳞状细胞癌、腺癌和大细胞癌等。在国内外关于肺癌脑转移危险因素的研究中，多数研究^[22, 25-31]^均表明肺腺癌或非鳞癌是NSCLC患者发生脑转移的独立危险因素。基于SEER数据库分析，大细胞癌的脑转移发生率最高（12%），鳞癌的脑转移发生率最低（6%）^[15]^。有研究^[28]^也得出了相同的结论，多因素分析显示非鳞癌患者脑转移明显高于鳞癌患者（*P* < 0.01）。另有一些研究比较了腺癌和非腺癌患者脑转移的风险。Zhang等^[22]^的研究纳入了637例的NSCLC患者，发现病理类型为腺癌患者的脑转移率是非腺癌患者的2.86倍（*P*=0.001），该研究还显示，低分化患者比高分化患者发生脑转移的风险显著升高（HR=1.91, 95%CI: 1.06-3.43, *P*=0.030）。有研究^[26]^进一步探索肺腺癌亚型与脑转移间的关系，通过分析332例I期-III期表皮生长因子受体（epidermal growth factor receptor, *EGFR*）突变型肺腺癌患者资料发现微乳头状型（micropapillary pattern, MIP）的肺腺癌患者脑转移发生率较高（MIP阳性组为56%，MIP阴性组为35.5%），且发生脑转移时间明显缩短，无病生存期分别为13个月和22个月（95%CI: 19.4-24.6, *P* < 0.001）。有研究^[24]^复阅了97例发生脑转移以及190例未发生脑转移的肺腺癌患者苏木精-伊红（hematoxylin-eosin, HE）染色标本切片，发现脉管瘤栓、微乳头型、坏死是肺腺癌脑转移的重要危险因素。脉管瘤栓和微乳头样改变使脑转移的危险性分别增加2.501倍（*P*=0.009）和2.128倍（*P*=0.024）。局灶性坏死与广泛性坏死组织患者与无坏死组织患者相比脑转移风险分别增加5.227倍和8.713倍（*P* < 0.001），同时，该研究还发现坏死程度越高，脑转移的时间越短。综上所述，在NSCLC患者中，非鳞癌或腺癌患者的脑转移发生率要高于鳞癌患者。可能的原因为腺癌以浸润性生长为主，发生血行转移的机会相对较高，容易发生脑转移，而MIP的存在增加了癌细胞侵入血管的能力，肿瘤的高增殖率导致肿瘤细胞乏氧坏死，引起与乏氧和肿瘤细胞血管生成相关的基因上调，增加了脑转移的风险，缩短了脑转移的时间。故当NSCLC病理亚型为腺癌且有MIP或出现广泛坏死时，临床医生需要增加颅脑的随访频率。

### 1.5 治疗方式

#### 1.5.1 不可手术的患者

对于根治性放化疗的NSCLC脑转移患者高危因素相关研究较少。目前研究^[32, 33]^均显示对于根治性放疗的患者，无论是否化疗，脑转移的发生率均无显著差异，放疗的剂量与脑转移的发生也未显示出统计学差异。

#### 1.5.2 可手术的患者

手术方式是否影响NSCLC脑转移目前尚未明确。Wang等^[31]^纳入的223例III-N2期NSCLC患者中，不完全切除和完全切除患者术后3年脑转移率分别为64.7%和35%（*P*=0.001）。另一项研究^[30]^对比了323例肺叶切除术及34例全肺切除术5年脑转移率，分别为35.6%及27.3%，但未见统计学差异（*P*=0.798）。其他几项研究^[16, 18]^同样未发现手术方式显著影响NSCLC脑转移。目前仍需更多的研究探讨手术方式与脑转移风险的关系。

更多的研究关注了新/辅助化疗对脑转移风险的影响。大部分研究显示系统化疗可降低颅外转移的概率，而脑转移发生率相对增加。一项回顾性研究^[25]^发现，cN2患者接受新辅助化疗与未接受新辅助化疗的患者脑转移的总发生率分别为32%和18%（*P* < 0.05），其中孤立脑转移的发生率分别为22%和11%（*P* < 0.05）。另一些研究^[34, 35]^也得到相同的结论。有研究^[36]^还报道了行新辅助治疗后病理显示完全缓解的局部晚期NSCLC患者后续发生脑转移的风险极高，可达55%。有研究者进一步探究化疗方案对脑转移发生率的影响。一项研究^[28]^纳入107例IIIa期行新辅助化疗的NSCLC患者，发现TP方案（紫杉醇联合顺铂）与EP方案（依托泊苷联合顺铂）相比3年内脑转移发生率低（31% *vs* 52%, *P*=0.011）。Mamon等^[37]^同样认为非紫杉醇组更容易发生脑转移（*P*=0.044）。NSCLC术后辅助化疗与脑转移发生的研究较少。Liang等^[18]^对193例IIIa期NSCLC患者进行回顾性分析，发现术后辅助化疗与未行辅助化疗的患者的3年脑转移率分别为47.3%和30.5%，但未见明显统计学差异。Besse等^[38]^进一步探索蛋白水平进行错配切除修复蛋白1（excision repair cross complement 1, ERCC1）对行术后顺铂辅助化疗的非鳞癌患者脑转移发生率的关系，发现ERCC1阴性的患者中辅助化疗会增加脑转移的发生率，而在ERCC1阳性组中没有看到这一趋势。综上所述，化疗相对增加脑转移的发生率，原因可能是给予化疗的患者生存期更长，患者有更多的机会观察到脑转移发生^[31]^。应用紫杉醇类化疗药物治疗可降低脑转移风险，可能原因为应用的患者多为非腺癌患者，且与放疗联用可达到增敏的效果，从而延缓肿瘤的进展。

围手术期胸部放疗与脑转移发生率相关研究较为有限。有研究^[28]^发现给予术前放疗的患者较仅术后放疗的患者脑转移率降低，3年脑转移率分别为33%和48%（*P*=0.035）。而多数研究^[30, 37]^均未发现放疗是脑转移的影响因素。

## 2 分子生物学研究

### 2.1 基因突变和表达谱

#### 2.1.1 EGFR

近年来，研究者发现*EGFR*突变状态可能影响NSCLC中枢神经系统的进展。Stanic等^[39]^发现*EGFR*突变患者在诊断时的脑转移发病率呈上升趋势（19% *vs* 13%, *P*=0.078）。Hsu等^[40]^对543例NSCLC患者的分析进一步证实了*EGFR*突变患者与野生型患者相比，脑转移累计发生率有显著差异（39.2% *vs* 28.2%, *P*=0.038, HR=1.4）。一项*meta*分析^[41]^报道，*EGFR*突变型患者发生脑转移的概率是*EGFR*野生型的2.48倍（95%CI: 1.46-4.20, *P* < 0.01）。Shin等^[42]^证明*EGFR*突变型肺腺癌患者脑转移的风险增加（OR=3.83, 95%CI: 1.72-8.55, *P*=0.001），亚组分析提示对于可手术的NSCLC，*EGFR*突变的患者术后脑转移复发风险更高（HR=4.49, 95%CI: 1.20-16.80, *P*=0.026），并且*EGFR*突变型患者显示出广泛的脑损害，多发性脑转移瘤（≥2个转移病灶）在*EGFR*突变患者中的检出率明显高于*EGFR*野生型（78.6% *vs* 47.8%, *P*=0.022）。另一项研究^[43]^通过对比61例NSCLC患者手术切除的原发肺肿瘤和成对的脑转移标本发现*EGFR*（51%）、*TP53*（41%）和*KRAS*（18%）是NSCLC原发灶最常改变的3个基因；在*EGFR*突变患者中，外显子19缺失突变的患者更多，占56%，而L858R突变的患者占28%；同时该研究还发现脑转移灶存在异质性，*EGFR*外显子19缺失与原发灶的符合率为100%，而*EGFR*外显子21L858R突变的符合率较低，为71%。综上所述，*EGFR*突变在NSCLC的脑转移中起着重要的作用，外显子19缺失可能更易发生脑转移，可能是由于肿瘤分子存在异质性以及*EGFR*基因在发展过程中表现出的不稳定性引起的。目前EGFR引起脑转移的机制尚不明确，有学者发现通过MAP激酶激活EGFR-MET相关信号对NSCLC的侵袭和脑转移非常重要；也有学者提出STAT3-miR21途径的激活在肺-脑转移转化中起调节作用^[45]^；突变型*EGFR*可通过IL-6调节介导STAT3激活，导致肺腺癌通过复杂途径发生转移^[46]^。目前需要对信号通路和分子机制进行进一步的研究，以揭示EGFR和NSCLC脑转移之间的确切关系。

#### 2.1.2 间变性淋巴瘤激酶（anaplastic lymphoma kinase, *ALK*）

*ALK*是另一个对NSCLC患者具有重要意义的驱动基因。近年来，一些研究表明*ALK*融合突变阳性的NSCLC患者脑转移发生率高。Wang等^[47]^通过分析552例局部晚期的NSCLC患者发现*ALK*基因融合突变与脑转移相关（*P*=0.021）。在Profile 1007研究中，超过40%的*ALK*突变患者出现脑转移，远高于*ALK*未突变的患者（19.1%）^[48]^。这可能是由于ALK抑制剂明显改善了ALK阳性NSCLC患者的预后，目前相关机制尚未明确，期待进一步的基础研究。

#### 2.1.3 *SPOCK1*及*TWIST2*

*SPOCK1*基因位于染色体5q31区域IL9和EGR1片段之间，该区域内基因片段可编码多种神经递质及激素受体等^[49]^。*TWIST2*基因位于人2q37.3染色体上，编码160个氨基酸组成的TWIST2蛋白^[50]^。*SPOCK1*及*TWIST2*基因在恶性肿瘤（如肺癌、肾癌、黑色素瘤、结直肠癌、乳腺癌）中呈高表达，与肿瘤的发生、转移相关，是目前的研究热点。有研究^[51]^在NSCLC脑转移患者身上获取脑转移组织并培养出具有干细胞性的细胞，称为脑转移启动细胞（brain metastasis-initiating cells, BMICs），通过建立异体移植模型，对BMIC模型进行体内和体外RNA干扰筛选，最终确定了*SPOCK1*和*TWIST2*是重要的BMIC调节基因，促进肿瘤从肺到脑的转移。同时该研究对比12例发生脑转移以及14例无脑转移NSCLC患者的原发灶组织标本进行验证，发现SPOCK1和TWIST2的表达仅在最终发展为脑转移的患者的原发性肺癌标本中观察到（SPOCK1: *P* < 0.01; TWIST2: *P* < 0.000, 3），并且也存在于相应的患者的脑转移组织中。这项研究提示*TWIST2*和*SPOCK1*是调控肺癌脑转移的关键基因。

#### 2.1.4 其他基因

目前有研究^[2, 16, 52]^显示某些分子标记物如血管内皮生长因子A（vascular endothelial growth factor A, *VEGFA*）、*KRAS*、*Ki67*、caspase-3的表达可作为肺癌脑转移高风险的预测因子，对这些因子的进一步研究可以更好地指导治疗。

### 2.2 微小RNAs（microRNAs, miRNAs）

miRNAs是一类19 nt-25 nt的非编码小RNA，具有重要的调控作用。生物信息学研究预测30%的哺乳动物蛋白质编码基因可能受到miRNAs的调控。近十年，miRNAs在脑转移表型中的作用机制得到关注。Arora^[53]^比较7例脑转移和6例非脑转移的NSCLC患者原发灶的miRNAs表达谱，发现miRNA-328和miRNA-330-3p的表达能够区分脑转移与非脑转移患者，且在另外15例患者中进行验证，其中12例验证成功。另一项研究^[52]^发现NSCLC患者原发肿瘤组织和周围正常肺组织相比，脑转移组的原发灶和脑转移灶中miRNA-375均下调。此外，miRNA-375在脑转移灶中的表达低于该患者的原发灶。Lu等^[54]^提取147例存活5年的无复发NSCLC患者和25例脑转移的NSCLC患者手术标本中的miRNAs进行分析，发现了10个与脑转移相关的miRNAs（hsa-miR-450b-3p、hsa-miR-29c^*^、hsa-miR-145^*^、hsa-miR-148a^*^、hsa-miR-1、hsa-miR-30d、hsa-miR-187、hsa-miR-218、hsa-miR-708^*^和hsa-miR-375）。Sun等^[55]^发现miR-4270下调和miR-423-3p上调与肺腺癌患者脑转移风险增加有关。这些研究均证明miRNAs与脑转移有密切关系，但样本量较小，且缺乏完整的机制研究，进一步探寻miRNAs在NSCLC患者脑转移发生中的作用是将来研究的重点。

## 3 NSCLC脑转移预测模型

### 3.1 临床因素预测模型

目前关于NSCLC患者脑转移预测模型多为临床因素相关预测模型。Sun等^[56]^对352例NSCLC患者进行分析，多因素*Cox*回归分析筛选出肿瘤原发灶-淋巴结-转移（tumor-node-metastasis, TNM）分期、病理类型、分化程度、淋巴结转移和血清NSE水平这5个影响NSCLC发生脑转移的独立因素用于构建nomogram模型，一致性指数（C-index）为0.83（95%CI: 0.79-0.91）。有研究^[23]^纳入I期-III期NSCLC患者1, 218例，中位随访时间为43.6个月，脑转移率为8.5%，首次复发部位为脑转移发生率为7.1%。通过多因素分析最终确立了包括组织学、吸烟状态、pT分期与pN分期这4种临床因素的预测模型，可预测2年和5年首发脑转移的概率，该模型具有较好的风险预测效果，2年和5年的曲线下面积（area under curve, AUC）分别为69.3%（95%CI: 62.6%-76.0%）和69.8%（95%CI: 63.6%-75.9%）。Zhang等^[22]^回顾性分析了637例I期-III期的NSCLC患者，根据多因素分析明确了4个独立的危险因素：NSE、组织学类型、转移淋巴结数目和肿瘤分级，并建立了预测模型计算3年及5年的脑转移概率。近年来，研究者将基因检测纳入危险分型中，一项研究^[47]^根据多因素分析明确了EGFR、RET、ALK、淋巴结转移、年龄和病理类型这6个高危因素并绘制受试者工作特征曲线（receiver operating characteristic curve, ROC），AUC=0.705（*P* < 0.001, SE=0.017, 95%CI: 0.671-0.739）。Wang等^[31]^对III-N2期NSCLC患者进行分析，建立了脑转移的高危数学模型：logit(*P*)=8.215-0.903×纵隔淋巴结阳性数-0.872×手术性质-0.714×病理类型-1.893×纵隔淋巴结转移程度-0.948×病理分期-1.034×术后化疗，*P*≥0.44为脑转移高危，敏感度为80%，特异度为77%。Zhang等^[57]^用196例患者进行外部人群验证，发现该模型预测脑转移的灵敏度为84.3%，特异度为64.6%，阳性预测价值为63.6%，阴性预测价值为84.9%，一致性检验*Kappa*值为0.47（*P* < 0.001），证实该模型具有较好的预测能力。Li等^[58]^随后根据此数学模型筛选出脑转移高危患者入组III期PCI随机对照研究，结果表明高危组患者行PCI将5年脑转移风险由49.9%降低至20.3%（HR=0.28, 95%CI: 0.14-0.57, *P* < 0.001），与既往PCI研究结论一致；同时，该研究报道PCI组的DFS延长7.3个月（28.5个月*vs* 21.2个月，HR=0.67，95%CI：0.46-0.98，*P*=0.037）。由于研究提前终止，DFS的获益并未进一步转化为OS的获益（HR=0.81, 95%CI: 0.56-1.16, *P*=0.310）。但该系列研究仍为NSCLC/PCI研究带来曙光，是目前唯一一个通过筛选脑转移高危患者入组的III期临床研究，不但明确了对脑转移高危患者进行PCI可推迟脑转移的发生，而且有望转化为生存获益，同时进一步证实了PCI获益人群筛选的必要性。

### 3.2 分子生物学预测模型

Fu等^[59]^通过对7例脑转移患者和45例无复发转移的原发性肺腺癌标本进行RNA序列分析，探讨了与肺腺癌脑转移的相关基因。通过初步筛选，得到8个差异表达的基因（*CDK1*、*KPNA2*、*KIF11*、*ASPM*、*CEP55*、*HJURP*、*TYMS*和*TTK*）。对272例肺腺癌手术切除标本进行差异表达基因的免疫组化检测，最终建立了由5个基因（*TYMS*、*CDK1*、*HJURP*、*CEP55*和*KIF11*）蛋白表达水平组成的脑转移评分系统（brain metastasis scoring system, BMS）=1.147, 9×TYMS+0.566, 4×CDK1+0.474, 8×HJURP+0.273, 1×CEP55+0.225, 2×KIF11，BMS的最佳cut-off值为1.266, 4，该评分对脑转移有较高的预测能力（12个月AUC：0.791，36个月AUC：0.766，60个月AUC：0.812）。BMS对验证数据集GSE31210和GSE50081总生存率的验证也显示出良好的预测价值（GSE31210，12个月AUC：0.682，36个月AUC：0.713，60个月AUC：0.762；GSE50081，12个月AUC：0.706，36个月AUC：0.700，60个月AUC：0.724）。该研究为首个根据基因预测NSCLC脑转移的模型，且60个月AUC > 0.8，有较好的预测性；另外，研究应用两个验证数据集进行外部验证，进一步评估了模型的准确性。

### 3.3 预测模型研究中存在的问题

所有研究均在内部进行验证且表现出良好的预测能力，提示预测模型在NSCLC脑转移高危人群筛查中具有应用潜能，其中部分研究具有良好的风险预测效果，AUC≥0.8。然而，目前预测模型仍存在几个问题：①研究纳入人群、模型构建方法及纳入因素指标差别较大，各研究之间的可比性较差；②目前的预测模型仅有一项研究结合了临床因素及常见检测基因，多数研究仅为单纯临床因素模型或分子生物预测模型，随着精准医疗时代的到来，两者联合是必然趋势；③所有研究均为单中心回顾性分析，仅有两项研究在外部人群中进行了验证，应用推广可能受到一定限制。各模型中种族单一，对全球范围内高危病例筛选有一定局限。

## 4 小结

NSCLC患者具有较高的脑转移风险，既往研究报道肿瘤标志物、病理类型、原发肿瘤分期、辅助/新辅助治疗是脑转移的独立危险因素。随着分子生物学检测的发展，一些基因、通路和miRNA也相继被报道是预测脑转移的分子指标。通过筛选NSCLC患者脑转移的高危因素建立预测模型有助于开展个体化的患者随访和预防策略。但目前风险预测模型仍存在一定缺陷，随着人工智能及大数据时代的到来，综合海量临床及生物组学信息的预测模型必将成为新的研究热点。
